# Investigating music teachers' ICT skills and technical possibilities in the field of online music education during the COVID-19 pandemic

**DOI:** 10.1016/j.heliyon.2023.e16463

**Published:** 2023-05-28

**Authors:** Judit Váradi, Gabriella Józsa, Adrienne Sz. Fodor, Viktória Molnár-Tamus, Tímea Szűcs

**Affiliations:** a University of Debrecen Faculty of Music, Hungary; b Károli Gáspár University of the Reformed Church, Faculty of Pedagogy, Hungary; c Debrecen Reformed Theological University, Hungary; d University of Debrecen, Faculty of Arts, Department of Educational Sciences, Hungary

**Keywords:** Online music education, Art education, Music teacher, COVID-19

## Abstract

The effectiveness of music education depends on the personal interaction between teachers and students in the pedagogical process. The presence of the music teacher, the initial presentation of music, and the immediate correction are all essential in individual instrumental training and group-based music education [1]. In our study, we examined the ICT skills and technical possibilities which music teachers (N = 352) had at their disposal during the COVID-19 pandemic, compiled the Internet platforms they used in their teaching, and asked whether they produced their own teaching materials. By using factor analysis, we explored music teachers' attitudes towards online education and identified four factors, namely student-centred, digital virtuoso, digitally creative, and difficult-to-adapt factors. The change in the learning environment and in familiar methods presented new challenges to most surveyed music teachers, who were creative in adapting to the situation and in preparing suitable teaching materials for their students.

## 1 Introduction

Scientific research has often investigated questions of music pedagogy from the perspective of education theory. Before 2020, the infrastructure, participants, and possibilities of traditional music education were usually examined. A few years ago, efforts were launched to incorporate ICT tools in curricular and extracurricular music education. Before the emergence of online education, researchers compared the productivity derived from the use of digital tools with the characteristics of in-person music education. After the restrictions due to the pandemic were lifted, the focus turned to the features and effectiveness of online tools [2]. The improvement of IT infrastructure and the availability of tools do not automatically bring about a digital shift in education. Despite educational innovation and continuous software development, music education has not changed significantly in recent decades. During the lockdowns, Internet platforms of virtual space replaced the physical environment as the setting where teaching and learning occurred. The changed lifestyle and institutional context challenged music teachers from several perspectives at all levels of education. However, the difficulties faced by the community resulted in a major modernisation of teachers' methods, and the social challenge led to a change in education. New social media sites supporting remote education with creative ideas, online applications, and online exercises instantly became popular among music teachers, providing significant help in a situation when many teachers were left without guidance and lacked adequate preparation.

## 2 Theoretical background

### 2.1 Music education in traditional spaces

The importance of in-person connection during the process of music education and instrument lessons is a well-known factor. This interactive form offers a special experience for not only students but also music teachers, since individualised instrumental training allows for a high degree of mutual concentration [3,4].

Research findings show that the use of different methods and tools is a powerful source of motivation in music education. The active involvement in musical activities, the support of intrinsic motivation, the individual and communal experience of joyful music performance can be much more effective in music lessons with diverse methodologies [5,6]. In recent years, several digital games and tasks, which can be used effectively in music education, have appeared, yet they are seldom employed in the classroom. Today, students' environment in the family and at school inevitably features computers and smartphones. These students are members of Generation Z, who easily understand, learn, and later master and internalise the operation of digital tools. Based on the findings of numerous Hungarian and international research studies on online music education, conducted over the past year, we can conclude that a very close social alliance developed to keep education operational not only abroad but also in Hungary. This is the reason why the transition to digital remote learning was completed in a matter of days at the beginning of the pandemic [[7], [8], [9]].

### 2.2 Music education in online spaces

However, only teachers with previously acquired digital competences could keep up with online education, which was especially pronounced among older teachers [10]. Students, who are equipped with the necessary tools and have already mastered or are open and able to master self-regulated learning, are equally important participants in remote learning and represent a prerequisite for its effectiveness. Those involved in online teaching and learning – teachers, students, parents – could only follow the usual curriculum with significant additional time investment. It was mandated platforms and commercially widely available products which were used the most.

The inclusion and personalised support of students, ‘left out’ of online education, posed a significant challenge [11]. Traditional classroom learning turned out to be more effective than online education, whose otherwise considerable potential did not fully materialise [[12], [13], [14], [15]].

In recent decades, music education has not changed significantly, despite various ICT innovations in education. Although digital technology has been experimentally introduced in public music education, and suggest that it could be an effective resource for music teaching [16], classical instrumental education has kept its traditional methods.

The pandemic created an extraordinary situation, to which both music teachers and students adapted without prior experience. The traditional model of art education was supplemented by digital culture, which required a thorough rethinking of methodology but was ultimately successful. Applying already effective methods to the digital environment opened up new perspectives [17].

## 3 Research methodology

### 3.1 Aim of the research

The changing setting of music instruction represented unprecedented challenges to the music education community in Hungary. In November 2020, the Art Education Research Group investigated the effects of online art education and the related experiences, with the support of the Hungarian Academy of Arts Research Institute of Art Theory and Methodology. Our research aim was to explore the circumstances of online music education during the pandemic. The paper presents the music education process which was implemented remotely way during the pandemic Covid-19. The relevance of the study lies in the previously insufficiently researched importance of digital competencies for the students' ability to study online. The novelty of the research is in the shift of focus to the characteristics and effectiveness of online tools.

### 3.2 Demographic distribution

The target group of the research was made up of music teachers at the primary, secondary, and tertiary level. We chose convenience sampling to collect the data [18]. Accordingly, a link to the measurement tool was published on social media sites and was also sent to institutions via email. Responses were submitted between January and April 2021. A total of 352 respondents completed the questionnaire, with the over-representation of women (73%). The average age of music teachers who completed the questionnaire was 44 years, the youngest respondent was 21, and the oldest was 70. A significant majority of the interviewed teachers (81%) teach at elementary level, while 11% of them teach at secondary music schools and 8% at tertiary level in music academies.

On average, those interviewed have been working in the field for 21 years, the least being one year, but some have been working for 51 years. We also assessed how long the participants in the study have been teaching at their current workplace. On average, the respondent have been working at the institution where they are currently teaching for 14 years, the least of which is one year, and the longest time being for 46 years in the same place.

The distribution of music teachers' workplaces according to the type of settlement is shown in Table 1.

**Table 1:** The settlement of music teachers workplaces.

Settlement	Person	%
Over 1.000.000	49	14
51.000–99.000	98	28
21.000–50.000	148	42
6.000–20.000	19	5
up to 5.000	35	10

The majority of music teachers teach instrumental lessons, but we also find singing and music teachers at the elementary level, vocal teachers, piano accompanists, solfège and music theory teachers, choir and orchestra conductor, folk musicians and folk singers.

### 3.3 Research instruments

The creation of the questionnaire was preceded by two focus group interviews. The analysis of the responses was used to compile a self-developed measurement tool, consisting of 48 questions, including closed and open-ended questions, which focussed on the teachers' experiences regarding remote learning.

The questionnaire can be divided into two large parts – the first part contains the demographic background variables, and the second deals with the questionnaire about online education.

### 3.4 Music teacher attitudes towards online education

In the second part of the questionnaire, we included a series of 16 questions measuring music teachers' attitudes towards online education. We termed this the ‘digital attitude scale’. Respondents were asked to rate on a four-point Likert scale how much they agreed with each statement – *strongly disagree; disagree; agree; strongly agree.* After reversing negative statements, factor analysis was performed to test the validity of the questionnaire, with a Kaiser-Meyer-Olkin coefficient of 0.810, which allowed us to perform factor analysis as it was valid to look for subscales behind the statements. Based on the literature, factor weights above 0.4 were considered acceptable. The items with the highest factor weights in the resulting factors were selected (Table 1), which was obtained by Promax rotation.

Table 2 shows that the factor analysis isolated four subscales, explaining 55% of the variance for the elements of the digital attitude scale. We named the resulting factors with respect to digital attitudes – the first factor was named ‘Learner-centred’, the second ‘Digital virtuoso’, the third ‘Digitally creative’ and the fourth ‘Difficult to adapt’.

**Table 2:** Factor analysis of the digital attitude scale. *Source:* OME database 2021.

Statements	Subscales			
	Learner-centred	Digital virtuoso	Digitally creative	Difficult to adapt
Eigenvalue	4.436	1.791	1.390	1.178
Variance (%)	27.722	11.192	8.685	7.360
Cumulative variance (%)	27.722	38.915	47.599	54.959
1. I can create a safe, relaxed atmosphere for my students.	0.800			
2. I get on well with students in my classes.	0.739			
3. I am able to engage my students with a variety of tasks.	0.598			
4. I find it difficult to keep my students' attention in the online space.	0.582			
5. I can also motivate students well in the online space.	0.489			
6. I like challenges.		0.817		
7. I am open to new methods.		0.748		
8. I can easily adapt to new/changing circumstances.		0.705		
9. I can use IT tools easily.		0.636		
10. I can teach more effectively in the online space.			0.795	
11. I can motivate my students to practice at home more easily in online education.			0.775	
12. The lack of personal contact bothers me.			0.581	
13. I can easily provide a sense of achievement to students in online education.			0.444	
14. I find it exhausting to listen to the audio recordings sent by the students.				0.726
15. I do not like online education because of the additional administrative work.				0.710
16. Preparation takes more time for me.				0.632

The reliability of the subscales of the digital attitude scale obtained by factor analysis is presented in Table 2. Based on the literature [18], Cronbach’s α values above 0.6 were considered acceptable for psychological scales.

Table 3 reveals that the Difficult to adapt subscale had a reliability value below 0.6, presumably because of the small number of statements (3). As this subscale covered negative attitudes towards online education, we decided to include it in further analysis despite the reliability score being slightly below the threshold.

**Table 3:** Reliability of the digital attitude scale (Cronbach’s α). *Source:* OME database.

Scales	Learner-centred	Digital virtuoso	Digitally creative	Difficult to adapt
Cronbach’s α	0.734	0.738	0.636	0.555

**Table 4:** ICT skills by age group. *Source:* OME Database 2021.

Age group (years)	ICT skills before online education	ICT skills in online education	Difference
21–30	3.7	3.9	0.2
31–40	3.4	3.8	0.4
41–50	3.2	3.7	0.5
51–60	3.0	3.6	0.6
61–70	2.9	3.6	0.7

## 4 Results

### 4.1 Digital competences and ICT equipment

Our first set of questions focused on the technical tools used in online education. We asked music teachers if they had previously used digital tools in their teaching, and if so, with what frequency. Of our respondents (N = 350), 19 had never and 51 had always incorporated digital tools into their teaching, but the frequency of use between the two extremes was more typical, since 156 people rarely, and 124 often use these tools. Most of the music teacher (216) use their own equipment, the school provided the ICT tools only for 82 teachers.

According to the data shown above, only one teacher indicated inability to reach students during the period of online education (in a small town with less than 5000 inhabitants, for an extracurricular group session), one teacher could reach a few students, and ten respondents could contact less than half of their students. Approximately 20% of the teachers surveyed (65) reported successful contact with 51–80% of their students during online lessons. The largest group (273) of music teachers were those who could keep in touch with (almost) all of their students.

The following question is closely related to the previous one. How often did music teachers teach live online lessons? The lack of technical equipment and adequate Internet connection meant that 6.57% of the respondents (23) never taught any live online music lessons. Nonetheless, 32.56% (114 people) taught live sessions often or sometimes, with the majority (60.85%, 213 people) adapting to the situation and continuing with regular lessons.

### 4.2 Supportive environment

**Fig. 1:**
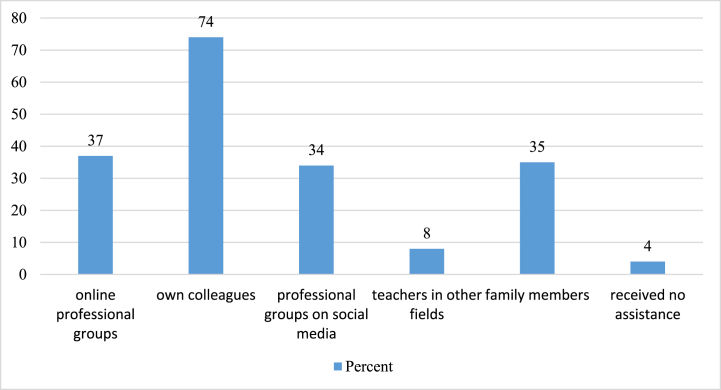
Assisting colleagues and communities in online education. *Source:* OME Database 2021.

Our questionnaire asked respondents about where they could get help from in relation to online education. Multiple answers could be selected. The data in Fig. 1 below illustrate how knowledge sharing within the institution was prominent, most respondents received support from their own colleagues (74%, 260 respondents). A similar proportion were assisted by online professional groups (37%, 130 people), professional groups on social media (34%, 119 people), and their family members (35%, 123 people). Only 13 colleagues reported that there had been no one to ask for help. The ‘other’ option was chosen by 16 respondents, but it was not clear whether they had turned to others for help or had suitable digital skills and needed no assistance.

**Fig. 2:**
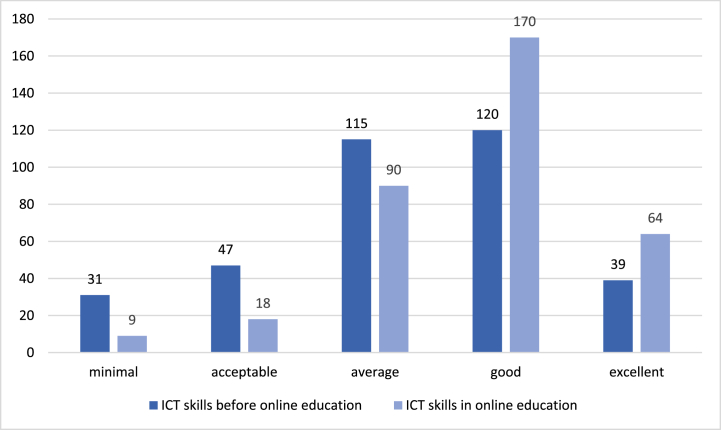
Music teachers' level of technological skills. *Source:* OME Database 2021.

One question of our survey related to music teachers' digital and technological skills. Referring to the period before the introduction of online education in March 2020, many people rated their computer skills as average (33%) or below (22%). At the time of completing the questionnaire, nine people still reported minimal skills, but the number of people who rated their own skills as excellent increased from 39% to 64% (Fig. 2). The most significant changes occurred for average and good skills.

As to skills prior to online education, we found that the oldest age group had the lowest self-assessment of digital skills (with a mean of 2.9). However, they also showed the biggest improvement – at the time of completing the questionnaire, they had an average skill level of 3.6, 0.7 higher than the previous one, which is comparable to the pre-pandemic levels for people aged 21–30.

The youngest respondent was 21 years old and the oldest was 70. As a first step, we created terciles by age. The first tercile comprised music teachers aged 21–38 years (n = 100), the second tercile music teachers aged 39–49 years (n = 94) and the third tercile music teachers aged 50–70 years (n = 107).

Second, we applied an analysis of variance to examine whether there were differences between age terciles in digital attitudes as verified by factor analysis (Table 5).

**Table 5:** ANOVA of digital attitudes and terciles by age. *Source:* OME database (N = 301).

	First tercile	Second tercile	Third tercile	F (p)
Learner-centred	0.024 (0.963)	0.120 (0.936)	−0.107 (1.079)	1.322 (0.268)
Digital virtuoso	0.156 (0.994)	0.002 (0.988)	−0.126 (0.991)	2.106 (0.124)
Digitally creative	−0.163 (0.965)	0.057 (0.977)	0.063 (1.016)	1.716 (0.182)
Difficult to adapt	0.038 (0.988)	0.080 (1.021)	−0.120 (0.977)	1.174 (0.311)

The analysis of variance found no statistical difference by age for any attitude, even though the oldest age group (third tercile) had the lowest means for all but the digitally creative attitude.

Respondents were divided into two groups based on their years spent teaching in music education – the first group included teachers who had been in the profession for less than 21 years, and the second group consisted of those who had been in the profession for more than 21 years. The distinction was used to examine differences in digital attitudes through a two-sample *t*-test (Table 6).

**Table 6:** T-tests of the digital attitude scale and the binary indicator of teaching experience. *Source:* OME database (N = 321).

	Group 1	Group 2	t (p)
Learner-centred	−0.002 (0.962)	0.001 (1.043)	−0.038 (0.970)
Digital virtuoso	0.050 (0.997)	−0.059 (1.000)	0.993 (0.322)
Digitally creative	−0.099 (0.924)	0.105 (1.066)	−1.838 (0.067)
Difficult to adapt	0.118 (0.976)	−0.112 (1.011)	2.078 (0.039)

The *t*-test identified a significant difference in the ‘Difficult to adapt’ attitude by the time spent in the field.

## 5 Discussion

### 5.1 Music teachers' digital knowledge and devices

In distance education the teachers had to face with some problems, for instance the lack of digital devices and digital literacy among teachers and students [19].

As in most professions, continuous self-education is significant for music teachers. Skill development may be closely related to the specialisation but could also relate to additional competencies.

In our empirical analysis, we examined the section of our questionnaire which focused on the surveyed music teachers' digital knowledge and skills as users of digital technology. In March 2020, music teachers set up several groups on social media platforms, in which group members could share ideas, teaching materials, music sheets, video recordings, and other document to assist each other.

Initially, we asked whether the music teachers who completed the questionnaire had received any training in addition their degree which contributed to the improvement of their digital literacy. Some 54% (189) of the respondents had received such training, indicating that they started online music education with relatively solid ICT knowledge.

It was generally difficult to suddenly adapt to a rapidly changing and highly unusual situation, in which the music teacher community was looking for ways to apply familiar in-person teaching methods in new and often unfamiliar settings.

In our research, we assessed the frequency of use of digital devices and inquired about their owners. According to the results, the most common solution in online music instruction was the use of teachers' own equipment. The question did not specify whether the respondents had already owned the devices or had to purchase them specifically for online teaching, but we assume that the immediate lockdowns led to inadequate device availability for many.

### 5.2 Characteristics of online education

Different educational circumstances, technical shortcomings, and families confined in their homes created new situations. For some, the relaxed environment of in-person instrumental training was replaced by a family setting which during the session was disruptive rather than supportive. Others, in contrast, welcomed the unfamiliar situation, which allowed teachers and students to get to know each other better. Some students were eager to show their teachers their home environment, toys, and pets, and during the idle hours at home they practiced on their instruments with joy, eagerly awaiting the next lessons.

Fig. 1 shows who the respondents asked for help during the remote education. They mostly received help from their own colleagues, but the role of online professional groups, social media and family members is also significant. Some music teachers enriched their knowledge with help from teachers of other disciplines. 13 people were left alone, they had was no one to ask for help. This number, although not high, is thought-provoking given that these respondents, who were mostly in their active years (21–55 years), had no one among their friends, colleagues, or acquaintances from whom they could obtain ideas and help to solve their problems. There were also some who turned to family members because they needed ‘someone to hold the mobile phone at the right angle to make an instructional video.’

According to the result of Fig. 2, for several teachers self-training and a strong improvement of digital skills helped achieve professionalism and high standards of teaching in a digital environment.

It is worthwhile to examine teachers' self-evaluation by age group. The five-point scale of the questionnaire corresponds to school grades, so that the averages for each age group are easy to interpret.

There was a negative association between self-reported digital skills and age, but the difference before and during online education increased with age. This can be explained by the regular use of digital skills, which in turn led to the acquisition of more proficient user skills, interest, and curiosity. At the same time, it is puzzling why young music teachers rated their own ICT skills so low. It may be necessary for teacher training institutions to alter the methodological curricula offered to students in order to help them become better equipped in this essential area by the end of their training.

### 5.3 Methodology of remote music education

We examined the frequency of live online classes. 6.57% of respondents could not teach due to technical reasons. In such cases, teachers sent their students various study materials (e.g., songs, music listening materials, playful music exercises) via the online platform used by the school. However, the majority were able to attend classes regularly.

Once the digital platform was chosen, its independent use and the organisation of the actual education for each session raised new questions. Within each institution, there was usually a colleague who could be approached for help with technical problems. However, the production of learning materials for music lessons required specific methodological assistance, ideally provided by music teachers with the same or similar specialisation supporting each other.

During in-person education, digital tools and musical materials are mostly used as a supplement to traditional instruction. However, during the pandemic, it was necessary to create novel digital content in the online space in addition to the usual teaching materials. It is beneficial to have teaching materials that teachers can apply with confidence, but the COVID-19 pandemic also highlighted the need for methodological innovation, whereby tasks are adjusted to the situation and to teachers' creativity, technical knowledge, and possibilities. The transformation to online education will effectively enable the development of more innovative and quality education [20].

Our results harmonise with the arguments of Alam and his colleagues [21] research, namely that during the lockdown the online education has led to changes in various components of the education system, such as the curriculum as well as the teaching and learning methods. We found that 143 music teachers (40% of our respondents) created new digital learning materials for their students. We list a few examples as illustration – videos to assist the learning of certain pieces, music history summaries, explanatory and tutorial videos on YouTube (e.g. for learning chord progression or reed carving), piano accompaniment for wind instrument players, exercises to compose melodies, listening exercises, tests, worksheets, films, folk music performances, recordings of a particular voice in polyphonic music, videos of exam concerts.

According the result of Table 4 those who had taught music for more than 21 years reported more difficulties in adapting to online education than those who had been in the field for less than 21 years.

### 5.4 Factors influencing the teacher's attitude

However, the extent to which teachers were able to maintain their students' motivation (χ^2^ = 2.563 p = 0.633) and interest (χ^2^ = 10.009 p = 0.264) in online education did not differ from the relevant teaching experience.

We examined whether the size of the settlement where the music teachers works results in a difference in attitude. The size of the settlement was divided into three categories – 1) big city (population of 51.000–1.000.000), 2) medium-sized city (21.000–50.000), 3) small city (2.000–20.000). The results of variance analysis show that there is only a difference in the student-centred attitude (F = 5749 p = 0,004) based on the size of the settlement. Student-centeredness is more present in larger settlements than in smaller ones. In other attitudes, there is no difference between the size of the settlement.

We expected to find significant differences in attitudes towards online education and its perceived effectiveness by age group. In other words, we hypothesised that older age groups would have more difficulties when adapting to online education and would thus be less successful in maintaining students' motivation and interest in online education, but this hypothesis was only partially corroborated. This is because we found no difference in digital attitudes by age, although we identified a significant difference in the difficulty of adapting to online education by comparing music teachers with more or less than 21 years of experience. Neither the literature nor this study can provide an unambiguous view on the relationship between age and attitude to online education. Furthermore, we found no differences by age and teaching experience in motivation or in the ability to maintain interest, which can be regarded as a performance indicator.

## 6 Limitation

Our research has examined the effects and experiences of online art education. Our self-developed questionnaire has focused on music teachers' distance education experience. The sample of the research was represented by colleagues involved in music education. We did not manage to get all the targeted music teachers to answer, for various reasons. We conducted our survey during the second wave of the pandemic and the questionnaires were filled out during the third phase. In this situation we did not reach all potential respondents, and many of them did not answer despite having been addressed. Our findings regarding the instrumental lessons lack the names of the students' specific instruments, because our questionnaire does not contain questions about this, and we feel this was not relevant for our research.

## 7 Conclusion

An important objective of art pedagogy is to bring about the renewal of art education in public education and of music education in particular. The novelty of this research is in the shift of the focus to the characteristics and effectiveness of online tools. Our research has showed that a possible direction for this renewal was uncovered through the widespread use of the online space. Similar to the research of Alsubaie [22], our results have revealed that the teachers' openness, creativity, and problem sensitivity were exemplary during the pandemic period. The continuous use of the online space contributed to the improvement of digital skills. Additionally, music teachers often tried to adapt to the changed circumstances by creating new educational content that can also be repurposed for teaching in the classroom. According to the results of the research, however, musical experiences, individual instrumental learning, and music lessons for groups can still be delivered most effectively in person.

Another significant result of our research has been the development of the digital attitude scale specific to music teachers. We investigated among music teachers the effect of age, teaching experience, and the place of residence on the subscales of the digital attitude scale, created by factor analysis. Of the background variables examined, outcomes differed significantly only by the place of residence. In towns with less than 5000 inhabitants, music teachers were less likely to manage their students in the online space, which resulted in a certain reluctance to change and challenge. Our results have identified no significant differences by age and teaching experience.

## 8 Future directions

This research has focused on an unusual situation, which shifted the attention of music teachers towards the use of digital tools in education. As a continuation of the research, it would be worthwhile to assess the extent to which modern tools and developed online teaching materials help the face-to face education, which has been restored again in the recent period. In the course of a comparative analysis of the digital possibilities of the various art sectors, it would be beneficial to explore the new direction of art education.

## Funding

This work was supported by the Research Institute of Art Theory and Methodology, Hungarian Academy of Arts.

## Declaration of competing interest

The authors declare that they have no known competing financial interests or personal relationships that could have appeared to influence the work reported in this paper
